# The regulatory effects of p53 on the typical and atypical ferroptosis in the pathogenesis of osteosarcoma: A systematic review

**DOI:** 10.3389/fgene.2023.1154299

**Published:** 2023-03-29

**Authors:** Linfeng Wang, Su Pan

**Affiliations:** Department of Orthopedic Surgery, The Second Hospital of Jilin University, Changchun, China

**Keywords:** osteosarcoma, typical ferroptosis, p53 ferroptosis, atypical ferroptosis, lipid peroxidation, SLC7A11, MDM2 activation

## Abstract

**Study background:** As a rare condition, osteosarcoma affects approximately 3% of all cancer patients. Its exact pathogenesis remains largely unclear. The role of p53 in up- and down-regulating atypical and typical ferroptosis in osteosarcoma remains unclear. The primary objective of the present study is investigating the role of p53 in regulating typical and atypical ferroptosis in osteosarcoma.

**Methods:** The Preferred Reporting Items for Systematic Reviews and Meta-Analysis (PRISMA) and the Patient, Intervention, Comparison, Outcome, and Studies (PICOS) protocol were used in the initial search. The literature search was performed in six electronic databases, including EMBASE, Cochrane library of trials, Web of Science, PubMed, Google Scholar, and Scopus Review, using keywords connected by Boolean operators. We focused on studies that adequately defined patient profiles described by PICOS.

**Results and discussion:** We found that p53 played fundamental up- and down-regulatory roles in typical and atypical ferroptosis, resulting in either advancement or suppression of tumorigenesis, respectively. Direct and indirect activation or inactivation of p53 downregulated its regulatory roles in ferroptosis in osteosarcoma. Enhanced tumorigenesis was attributed to the expression of genes associated with osteosarcoma development. Modulation of target genes and protein interactions, especially SLC7A11, resulted in enhanced tumorigenesis.

**Conclusion:** Typical and atypical ferroptosis in osteosarcoma were regulatory functions of p53. The activation of MDM2 inactivated p53, leading to the downregulation of atypical ferroptosis, whereas activation of p53 upregulated typical ferroptosis. Further studies should be performed on the regulatory roles of p53 to unmask its possible clinical applications in the management of osteosarcoma.

## Introduction

As a bone tumor, osteosarcoma affects the origin of the mesenchyme and mainly occurs during the growth phase of long bones (Prater and McKeon, 2020; Liu et al., 2022). The tumors grow around the epiphyseal growth plates of the tibia of the femur. In most cases, osteosarcoma has been associated with gene disorganization, dysregulation of genes suppressing tumors and cell cycle, and chromosomal alterations in aneuploidy, alongside inadequate repair of the deoxyribonucleic acid. Osteosarcoma is either inherited or acquired at birth (Hameed and Mandelker, 2018). However, most cases occur due to gene mutation. In addition, ferroptosis plays a crucial role in developing osteosarcoma, like other forms of cancer. Ferroptosis unfolds *via* phospholipid damage and the over-production of reactive oxygen species (ROS) (Zou and Schreiber, 2020).

Even though osteosarcoma remains uncommon worldwide, it raises serious health concerns as it leads to loss of bones, pain, and physical support for body structures among children aged 13 to 16 (Petriceks and Salmi, 2019). According to Petriceks et al., osteosarcoma accounts for approximately 3% of childhood cancer and less than 1% of cancers diagnosed in the United States of America in a year. Despite the small number of people affected, it is still necessary to understand the pathogenesis of osteosarcoma, which may be beneficial for the management of clinical treatment guidelines.

The genetic material is also associated with the atypical and typical ferroptosis in osteosarcoma. However, genetic outplay concerns target genes and mutations. For example, genes modulated by p53 or p53 target genes have been reported to play a significant role in the course of osteosarcoma *via* typical and atypical mechanisms (Rickel et al., 2017; Czarnecka et al., 2020).

**FIGURE 1:**
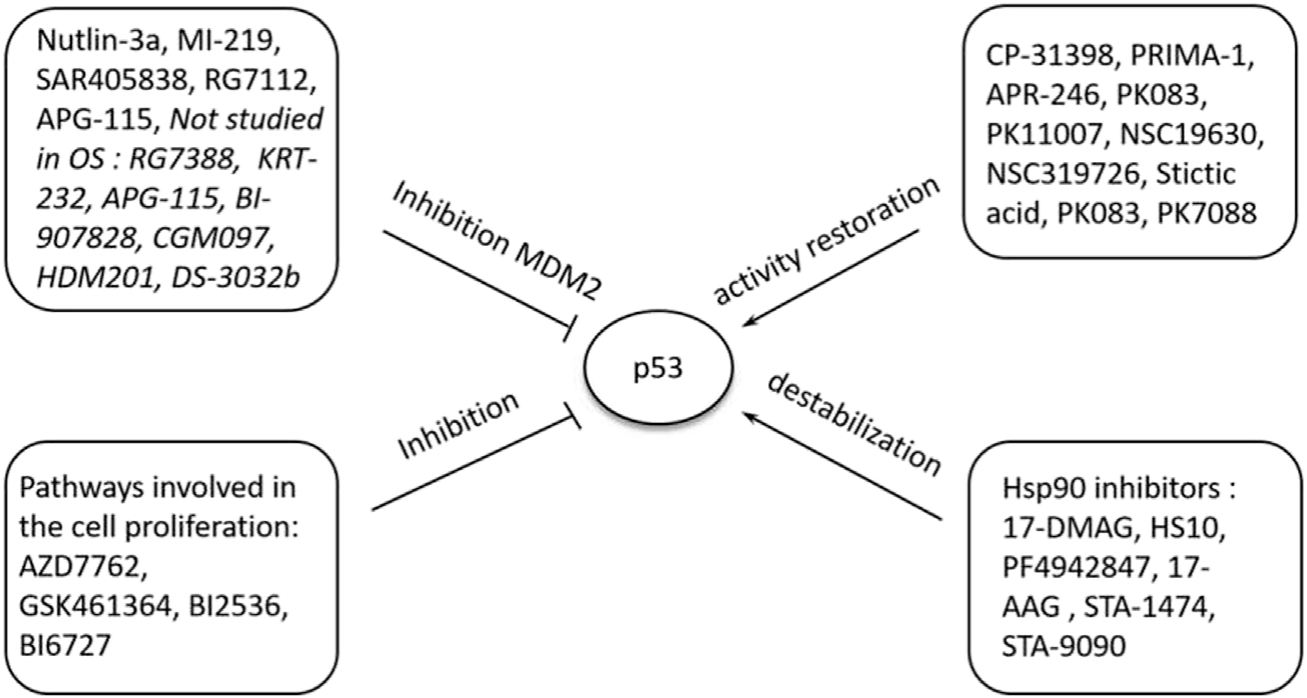
A summary of the regulation and occurrence of ferroptosis in osteosarcoma. A four‐fold manifestation of p53 shows how osteosarcoma outcomes unfolds: inhibition, destabilization, activity restoration and inhibition via MDM2. Each of these pathways down- and up‐regulate p53’s inhibitory and excitatory functions in osteosarcoma development.

The exact cause of osteosarcoma remains unknown. However, various biological processes and events have been associated with its pathogenesis. Of the biological elements attributed to osteosarcomas, p53 has been linked to different cellular processes and events. Previous literature has shown the role of p53 in regulating atypical and typical ferroptosis, where iron-dependent cell lysis is commanded by oxidative phospholipid damages (Zou and Schreiber, 2020). Cell death mechanisms stand out in understanding the role of p53 in osteosarcoma-related ferroptosis. Figure 1 illustrates the roles of p53 in atypical and typical ferroptosis in the course of osteosarcoma. TP53 mediates the expression of SLC7A11C in human cancers, and Liu et al. have clarified crucial elements concerning the roles of p53 in regulating atypical and typical ferroptosis in osteosarcoma (Liu et al., 2020). Moreover, previous literature has reported and emphasized the role of p53 in ferroptosis: up- and downregulation of atypical and typical ferroptosis.

**FIGURE 2:**
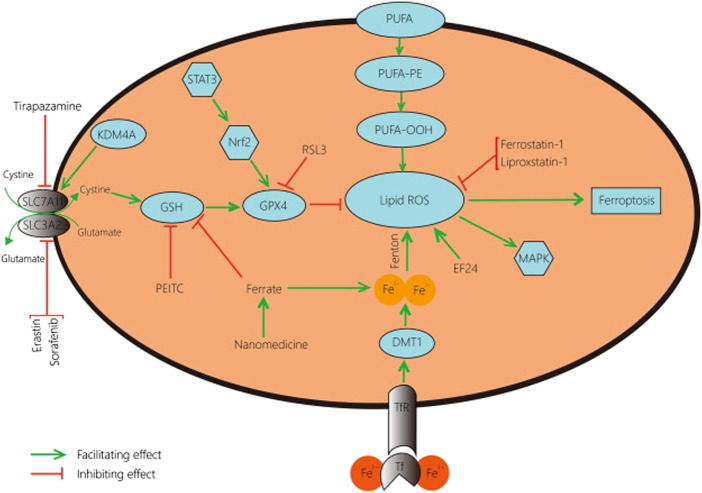
A schematic representation of typical ferroptosis mediated by P53. The outcome of p53-mediated ferroptosis is apoptosis; cell death mechanisms characterizing osteosarcoma.

In the present study, we focused on a systematic review of studies reporting the regulatory roles of p53 in atypical and typical ferroptosis in the tumorigenesis of osteosarcoma. Up and down-regulatory functions as discussed with a focus on how either of the two aspects influence osteosarcoma. In addition, this review investigated up- and down-regulatory functions performed by p53 in osteosarcoma (Zhao et al., 2021). Even though existing literature has demonstrated the regulatory roles of p53 (see Figure 1; Figure 2), more knowledge is required to expand an understanding of p53’s roles in ferroptosis in osteosarcoma.

Typical and atypical ferroptosis in osteosarcoma are complex biological processes (Figure 2). In the present study, we aimed to review and simplify evidence to enhance comprehension. Additionally, facts and evidence found in the present study were of great clinical significance. The target genes and interactions are potential clinical information for interventions against the disease and management (Giaccone, 2019; Chen et al., 2022). Robust outcomes will improve clinical outcomes of osteosarcoma management as better intervention approaches are developed.

## Methods

### Study design and database search

The initial literature search was performed in six electronic databases, such as EMBASE, Cochrane library of trials, Web of Science, PubMed, Google Scholar, and Scopus Review, following the Preferred Reporting Items for Systematic Reviews and Meta-Analysis (PRISMA) protocols (Page et al., 2021; Rethlefsen et al., 2021). Eligible studies were collected in the initial literature search. A specific approach was used to obtain the most appropriate studies matching the profile of the required studies. Eligible studies were identified by a rough view of the abstracts and titles.

### Search strategy

Keywords and Boolean operators were the primary tools used to identify eligible studies. Different keywords were used to locate the articles in the electronic databases. As mentioned, the initial literature search was performed in six electronic databases, and the keywords were indiscriminately applied to identify available material. The Boolean operators “AND” and “OR” were used to combine keywords (Table 1). Furthermore, the Boolean operator “AND” was used to combine keywords with distinct meanings, whereas the Boolean operator “OR” was used to combine keywords with similar meanings. Table 1 summarizes the keywords used to locate eligible literature in the present study.

**TABLE 1:** Search strategy: Electronic databases where literature search was done and the keywords.

Electronic database	Keywords	Regulatory effects on atypical or typical ferroptosis
EMBASE	Typical ferroptosis, atypical ferroptosis, p53	Upregulation
Cochrane Library of trials	P53, osteosarcoma, tumorigenesis, lipid peroxidation	Upregulation
Web of Science	P53 inactivation, p53 activation, typical ferroptosis	Dow-regulation
PubMed	P53, osteosarcoma, tumorigenesis, SLC7A11	Downregulation
Google Scholar	Mutant-p53, osteosarcoma, p53 target genes	Upregulation, downregulation
Sopus review	Mutant-p53, typical ferroptosis, atypical ferroptosis	Upregulation, downregulation

### Eligibility criteria

Study eligibility was determined by the Cochrane collaboration framework of inclusion and exclusion criteria (Cumpston et al., 2019; Campbell et al., 2020). PICOS protocols were used to assess eligible studies for inclusion in the review. However, since the present study did not rely on intervention and the outcomes, we only focused on patient features because these features would describe the role of p53 in ferroptosis in osteosarcoma.

### Inclusion criteria

1. Studies meeting the following criteria were included in the present review.
2. Patients: The patient population was adequately defined, per the requirements of the Cochrane Collaboration of systematic reviews. The present review included osteosarcoma patients only. They could be adults or children.
3. Intervention: Not applicable
4. Comparison: Not applicable
5. Outcomes: We focused on the role of p53 in typical and atypical ferroptosis among osteosarcoma patients. All study outcomes pertaining to these outcomes were observed and recorded. Studies reporting these outcomes were included in the present work.
6. Studies: All the studies included in the present review were PICO studies: studies reporting the role of p53 in ferroptosis among osteosarcoma patients.

### Exclusion criteria

Studies were excluded in the present review if they met the following exclusion criteria.

1 Studies reporting outcomes irrelevant to the present topic.
2 Non-PICO studies not reporting the regulatory roles of p53 in typical and atypical ferroptosis in osteosarcoma.
3 Ongoing studies.
4 Studies involving interventions that could distort or influence the outcomes negatively.

### Evaluation of quality of literature

The quality of the literature set for inclusion was assessed using Version 2 of the Cochrane risk of bias tool (Cochrane, 2011). The five domains of risk of bias were assessed: random sequence generation, allocation concealment, blinding of participants and personnel, incomplete outcome data, selective reporting, and other forms of bias, and the overall outcomes were reported as low risk of bias, high risk of bias, and unclear risk of bias. This quality assessment provided an image of the overall status of the quality of included literature. Furthermore, the quality of individual studies was assessed.

### Data selection and extraction

Data selection and extraction in the present review were performed by two independent reviewers. A systematic approach was deployed where the independent reviewers would examine the studies for potential information or data pertinent to the current topic. The data selection and extraction process were governed by discussion, as the independent reviewers occasionally disagreed on the type of data or particular information to be included in the present study. Disputes would be amicably solved through discussion, and data selection and extraction on studies reporting the role of p53 in atypical and typical ferroptosis in osteosarcoma were limited by the independent reviewers. These study outcomes would be reviewed to reveal the overall effects of p53 on atypical and typical ferroptosis in osteosarcoma.

## Results

### Study selection

**FIGURE 3:**
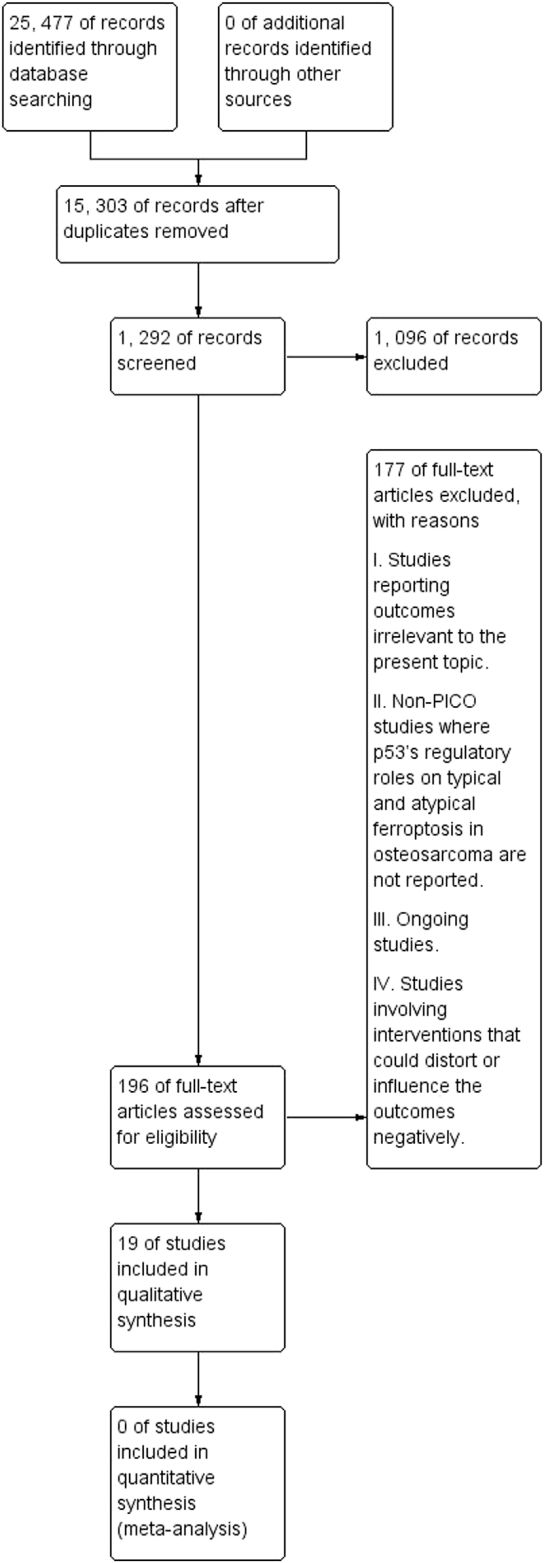
A PRISMA flowchart of the initial literature search.

Again, the study selection procedure was systematically performed by the independent reviewers. First, the independent reviewers checked the titles and abstracts of studies for eligibility. Studies with topics and abstracts unrelated to the present topic were not subjected to further analysis. Abstract screening seconded title screening as the latter was a key indicator of potential studies. Finally, eligible studies were subjected to full-text screening to establish accounts of data pertinent to the present topic. Figure 3 illustrates the flowchart diagram of the initial literature search. The initial literature search identified 25,477 records from the six electronic databases. After removing duplicated studies, 15,303 records remained, out of which 1,292 were screened. Such screening led to removing 1,096 records, with reasons previously indicated.

### Baseline features of included literature

Table 2 summarizes the basic features of the included literature. Focusing on the regulatory effects of p53 on atypical and typical apoptosis in osteosarcoma-related ferroptosis, we investigated studies reporting such outcomes by assessing the effects of ferroptosis and regulatory mechanisms of p53. The regulatory mechanisms indicate whether a regulatory effect is atypical or typical. The summary included two distinct pieces of information obtained from the studies; regulatory effects of p53 on atypical ferroptosis or typical ferroptosis. Included literature could be identified through author ID and the year of publication. We did not find a study reporting the regulatory effects of p53 on typical and atypical ferroptosis.

**TABLE 2:** Basic features of included literature.

Study	Effects of ferroptosis in osteosarcoma	Regulatory mechanism of p53	Typical ferroptosis	Atypical ferroptosis
Ji et al. (2022)	Enhanced tumor progression	Enhancing cellular sensitivity to ferroptosis	Induced	NA
	Reduced tumor progression	Delaying the generation of cells for ferroptosis	Induced	NA
Lu et al. (2020)	Induction and suppression of osteosarcoma	Enhanced interaction between p53 and SLC7A11	Up and downregulation	NA
Luo et al. (2018)	The wild-type p53 mutant is downregulated	Up or downregulation of ferroptosis	Up and downregulated	NA
Velletri et al. (2016)	Enhances tumor progression	Amplified apoptosis, metabolism, and regulation of cell cycle	Upregulation	NA
	Establishment of bone tumor microenvironment components	Mutations in p53 result in aberrant transformations of mesenchymal stem cells	Upregulation of osteosarcoma	NA
Sciot (2021)	Differentiation of tumor cells	MDM2 overexpression blocks p53’s regulatory mechanisms	Upregulation of osteosarcoma	NA
Usman et al. (2020)	Promotion of cancer progression through autophagy	Drug resistance and mutations	Upregulation	NA
Magri et al. (2021)	Promotes tumorigenesis	The wild-type p53 mutant indirectly modulates (an inhibitory mechanism) xCT expression	Upregulation	NA
Hatina et al. (2019)	Abrogation of tumor suppression processes	Modification of transcriptome and alteration of epigenetic regulation	Down and upregulation	NA
Bouchalova et al. (2014)	Enhanced cell proliferation	Induction of p53-oncogenic functions	NA	Induced
Su et al. (2022)	Cell death	Modulating programmed cell death	NA	Induced
Babamohamadi, et al. (2022)	Increased apoptosis	Abnormal signaling pathways	NA	Induced
Gnanapradeepan et al. (2018)	Lipid and iron-dependent cell death	Enhanced ferroptosis: high production of ROS and initiation of the lipid peroxidation	NA	Induced
Xie et al. (2016)	Cell death	Production of lethal reactive oxygen species and lipid peroxidation	NA	Induced
Leroy et al. (2017)	Tumor progression and activation of tumor suppressor TP53	Activation of p53 and the initiation of tumor suppressor TP53	NA	Induced
Yang and Zhang (2013)	Antagonistic targeting of osteosarcoma target sites and downregulation of tumorigenesis	Genetic aberrations	NA	Induced
Saraf et al. (2018)	Activation of antitumor processes	Inactivation of p53	NA	Induced
Pang et al. (2020)	Enhance antitumor functions	Activation of TP53 antitumor activities	NA	Induced
Van Maerken et al. (2014)	Loss of tumor suppressor functions of the p53	Loss-of-functions mutations in TP53	NA	Induced
Komori (2016)	Cell death	Deletion of p53 and inactivation	NA	Induced

### Quality appraisal

**FIGURE 4:**
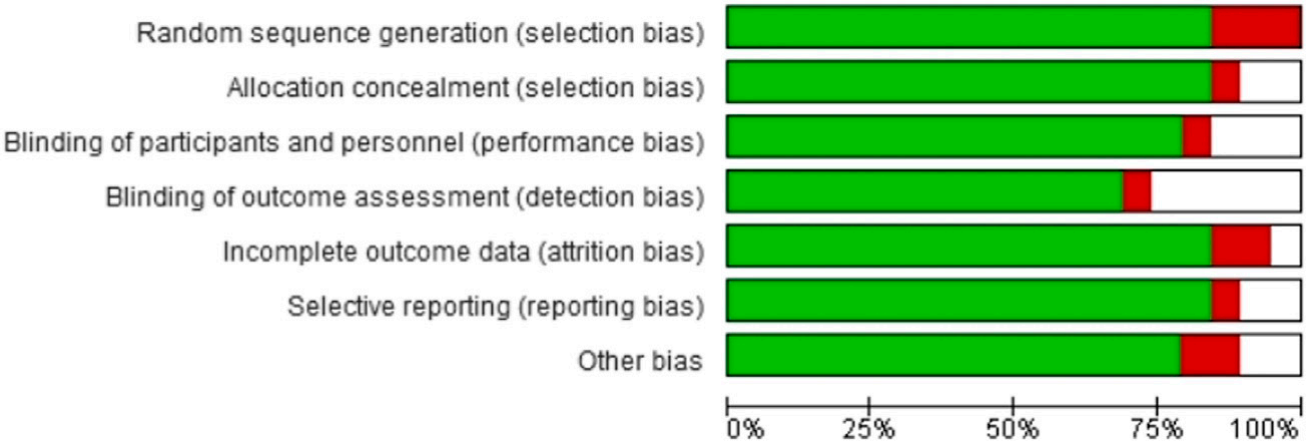
The overall risk of bias in included studies.

As indicated earlier, we summarized the overall risk of bias outcomes obtained from the Cochrane collaboration of systematic reviews (Version 2). Figure 4 summarizes the risk of bias in the seven domains, including random sequence generation, allocation concealment, blinding of participants and personnel, blinding of outcome assessment, incomplete outcome data, selective reporting, and other forms of bias. Color coding was used to represent the overall risk of bias in each domain. The green color represented a low risk of bias, whereas red and white color coding represented a high and unclear risk of bias, respectively. A visual inspection of Figure 4 showed that most studies had a low risk of bias.

### Quality assessments

**FIGURE 5:**
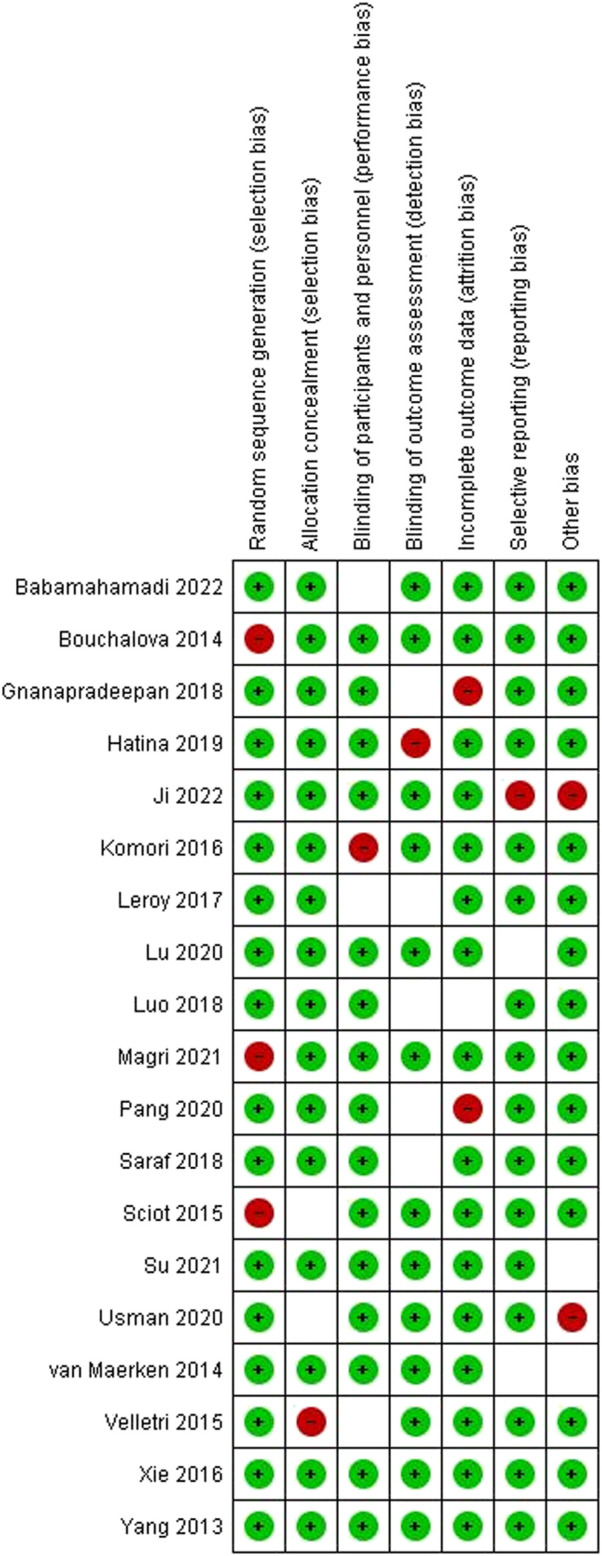
Risk of bias assessment outcomes on individual studies.

We investigated the quality of individuals to ascertain eligibility. Figure 5 represents the quality assessment outcomes of the studies in every domain. The color coding used in the figure above was applied in the present study. Color coding was used to represent the overall risk of bias in each domain. The green color represented a low risk of bias, whereas red and white color coding represented a high and unclear risk of bias, respectively.

### The regulatory effects of p53 on typical ferroptosis in osteosarcoma

We reviewed 10 studies to unmask the regulatory effects of p53 on typical ferroptosis in osteosarcoma and found different mechanisms and biological processes. Several studies (Velletri et al., 2016; Luo et al., 2018; Sciot, 2021; Hatina et al., 2019; Lu et al., 2020; Usman et al., 2020; Magri et al., 2021; Ji et al., 2022) have shown that p53 exerts different regulatory effects on typical ferroptosis, including enhancing cellular sensitivity to ferroptosis, delaying the generation of cells for apoptotic process, enhancing p53-SLC7A11 interaction, amplification of apoptosis, metabolism, and regulation of cell cycle, induction of aberrant mesenchymal stem cells (MSCs) through p53 mutations, MDM2 overexpression, drug resistance, indirect inhibitory modulation of xCT expression, induction of p53’s oncogenic functions, and transcriptome modification. The overall effect of these functions is up-regulating ferroptosis as they enhance p53’s functions in enhancing ferroptosis.

All these observations indicated that p53 modulated the pathway for typical ferroptosis differently. The mechanisms listed above emphasized the differences in regulatory measures reported by the studies. Therefore, the genetic regulatory role of p53 was crucial in the present review. Luo et al. (2018) have reported that the wild-type p53 exploits microRNAs to inhibit cancer development, and the gain-of-function mutant of p53 triggers oncogenic properties. Ji et al. have articulated the oncogenic properties and the pathways through which p53 regulates typical ferroptosis. According to Ji et al., p53 enhances cellular sensitivity to ferroptosis and functions like a rheostat, leading to the up- and downregulation of sensitivity. The mutation modifies p53, leading to loss of antitumor functions (Liu et al., 2019); P53 modification is the parent of the wild-type p53 protein and the loss of antitumor function. This is the mechanism through which ferroptosis is enhance.

**FIGURE 6:**
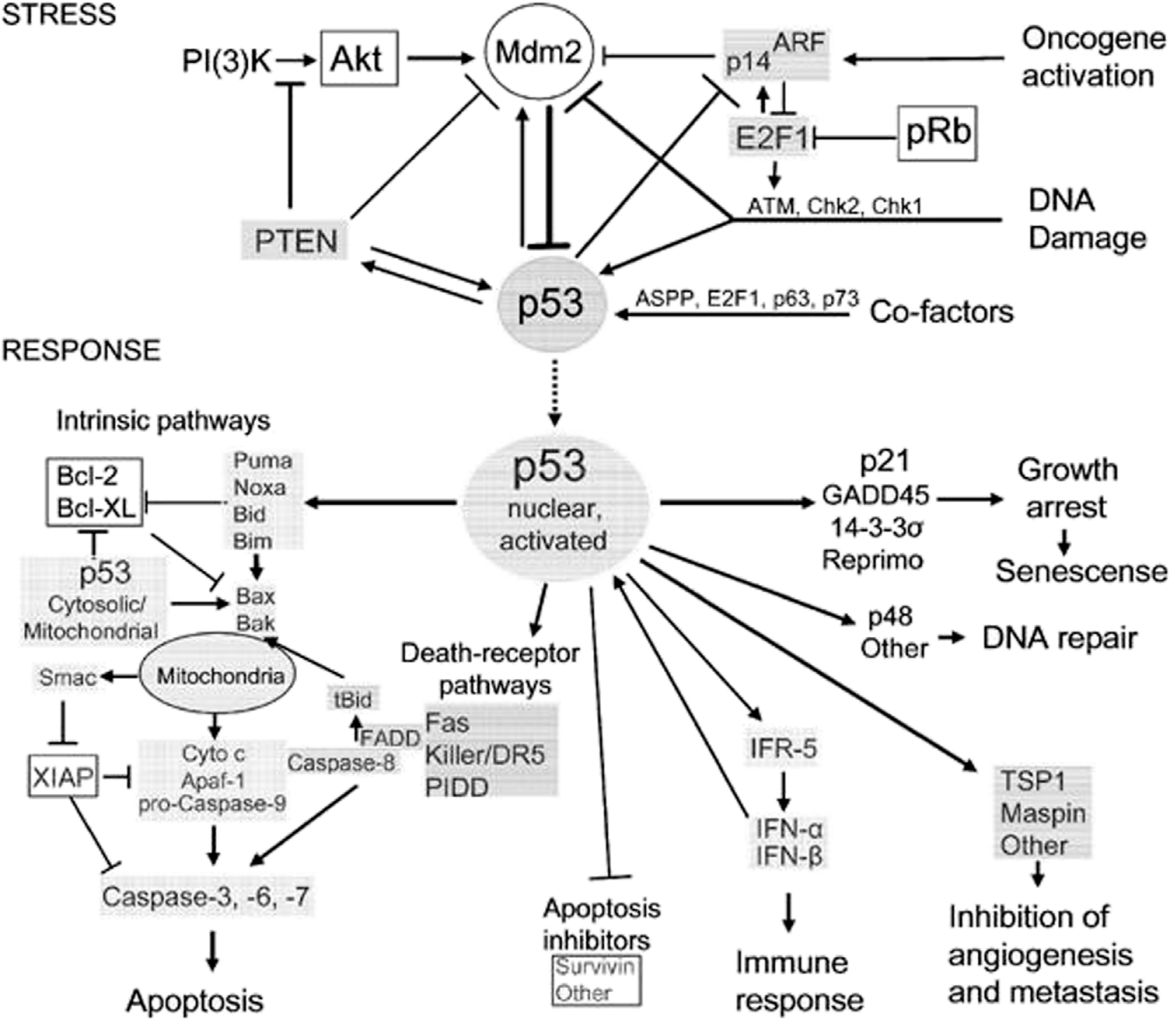
A schematic representation of p53’s role in modulation of typical ferroptosis in osteosarcoma. The resulting effects of the biological processes are based on molecular responses to stress. Nuclear activation related to p53 protein are generated from the stress induced by tumorigenesis.

The genetic approach to regulatory frameworks of p53 involves cellular susceptibility to ferroptosis. By enhancing the susceptibility of bone cells to ferroptosis, p53 inhibits cancer and the accumulation of mutations associated with osteosarcoma (Zhang et al., 2022a). Genetic mutations are at the center of p53-induced ferroptosis as genetic mutations affect the expression of cells, protein interactions, and the resulting outcomes. Figure 6 is a schematic representation of genetic modifications altering p53-PVT1 interaction and the effects on biological processes. The genetic modifications delay the generation of cells for ferroptosis (Ji et al., 2022), modulate SLC7A11-p53 interaction (Lu et al., 2020), cause up- and downregulation of ferroptosis (Luo et al., 2018), and amplify the transformation of MSCs and apoptosis (Velletri et al., 2016). Table 3 summarizes the roles of p53 in cell death characterizing ferroptosis in osteosarcoma.

**TABLE 3:** A summary of roles of p53 in different processes involved in osteosarcoma. Even though some of these processes do not unfold in typical and atypical ferroptosis, they are vital in explaining the roles of p53 in osteosarcoma.

Regulation of metabolism	DNA damage through oxidation	Cellular reprogramming
TIGAR, SCO^2^	ATR/CHK1 and ATM/CHK2	MMP-9
GLUT1 and GLUT4	SAPK, JNK and p38MAPK	CXC chemokines
PFKFB3 and PFKFB4		

### The regulatory effects of p53 on atypical ferroptosis in osteosarcoma

As the atypical tumor suppressor, p53 is produced under the instructions issued by TP53. We found that p53 regulated cell division through proliferation, which is rapid cell division and inhibiting uncontrollable cell growth (Kang et al., 2019). Gene targeting defines the regulatory effects of p53 on atypical ferroptosis in osteosarcoma. Liu et al. (2022) have reported that the upregulation of p53 directly inhibits the activity of SLC7A11, promoting ferroptosis. Therefore, we sought evidence of up- and downregulation of atypical ferroptosis and compared the outcomes. The role of p53 in regulating atypical ferroptosis could reduce the severity of the disease or implicate them by exacerbating tumor progression.

Upregulation and downregulation of p53 enhanced and reduced ferroptosis, respectively. Different studies have shown that atypical ferroptosis is either increased or decreased in osteosarcoma. Six studies (Bouchalova et al., 2014; Xie et al., 2016; Gnanapradeepan et al., 2018; Su et al., 2022; Babamohamadi et al., 2022) have reported that atypical ferroptosis is enhanced in osteosarcoma. Table 4 summarizes the regulatory mechanisms of p53 on atypical ferroptosis. We found that upregulation of p53 enhanced the production of ROS, medicated lipid and iron-mediated cell death, and increased proliferation. We compared these outcomes with p53 inactivation and the resulting status regarding osteosarcoma-related ferroptosis (Table 4).

**TABLE 4:** A comparison of p53’s up-and down-regulatory mechanisms in osteosarcoma-related ferroptosis.

Upregulation of p53		Downregulation of p53
Increased cell death	High production of lethal reactive oxygen species	Balancing the production of antioxidant cellular products to counter excess reactive oxygen species
Increased cell metabolism and cell death	Lipid and iron-mediated cell death	Down-regulating lipid peroxidation
Induction of p53 oncogenic functions	Enhanced cell proliferation	Activating p53’s antitumor functions and actibities

**FIGURE 7:**
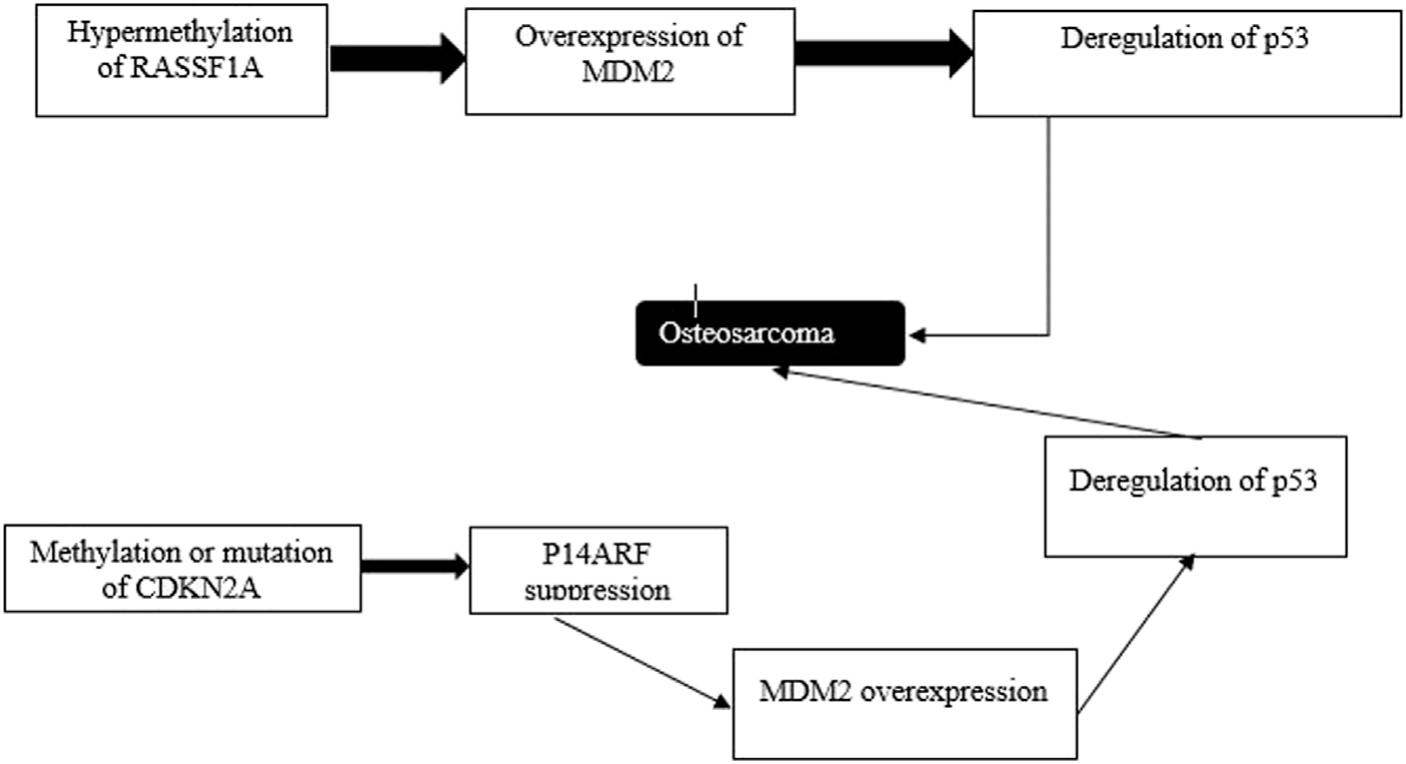
Atypical ferroptosis results from p53 deregulation. Epigenetic events contribute to the progression of induction of oxidation. Genes whose activities are modulated by p53 or p53’s target genes, including RSSF1A and CDKN2A are silenced *via* methylation.

Comparing the outcomes reported in Table 4 and the pathway illustrated in Figure 7, we could deduce two possibilities from the regulatory outcomes of p53: enhanced ferroptosis or inhibited ferroptosis (de Azevedo et al., 2019). Several studies (Bouchalova et al., 2014; Xie et al., 2016; Gnanapradeepan et al., 2018; Su et al., 2022; Babamohamadi et al., 2022) have indicated that upregulation of p53 enhances atypical ferroptosis in the course of osteosarcoma, whereas other studies (Komori, 2016; Van Maerken et al., 2014; Pang et al., 2020; Saraf et al., 2018; Yang and Zhang, 2013; Leroy et al., 2017) have reported that inactivation of p53 downregulates atypical ferroptosis in osteosarcoma.

### p53 and cell proliferation in osteosarcoma

We reviewed six studies reporting p53 inactivation and the resulting cell proliferation to illustrate the regulatory mechanisms of p53 in atypical ferroptosis in osteosarcoma. Several studies (Yang and Zhang, 2013; Van Maerken et al., 2014; Komori, 2016; Saraf et al., 2018; Pang et al., 2020;; Leroy et al., 2017) have reported the regulatory mechanisms of p53 on atypical ferroptosis in the course of osteosarcoma, while inactivation of the gene inhibits cell proliferation. These studies have shown that p53 downregulates atypical ferroptosis in osteosarcoma by inhibiting cell proliferation. In addition, Pang et al. (2020) have reported that 95% of osteosarcoma cases are associated with p53 inactivation.

However, we found unique and distinct mechanisms through which p53 inactivation implicated inhibitory effects on cell proliferation. Eventually, the inactivation of p53 inhibited the expression of SLC7A11 as the interaction between SLC7A11 and p53 was limited. We found that p53 inactivation resulted from multiple and different cell processes. Table 5 summarizes the different mechanisms reported by individual studies. The inactivation of p53 promotes the survival of tumor cells (Van Maerken et al., 2014). Leroy et al. (2017) and Pang et al. (2020) have shown that a high incidence of osteosarcoma is associated with TP53 mutation.

**TABLE 5:** A summary of mechanisms of p53 inactivation in the course of osteosarcoma.

Study	Reported mechanism of cell proliferation inhibition
Komori (2016)	Inhibition of cell cycle, induced cell death, inhibition of DNA repair, and antagonism of osteoblast proliferation
Van Maerken et al. (2014)	P53 regulation by MDM2. MDM2 activation inactivates p53, resulting in a decrease in p53 activation
Pang et al. (2020)	TP53 mutation
Saraf et al. (2018)	P53 mutation
Yang and Zhang (2013)	Apoptosis and tumor proliferation
Leroy et al. (2017)	TP53 alterations

**FIGURE 8:**
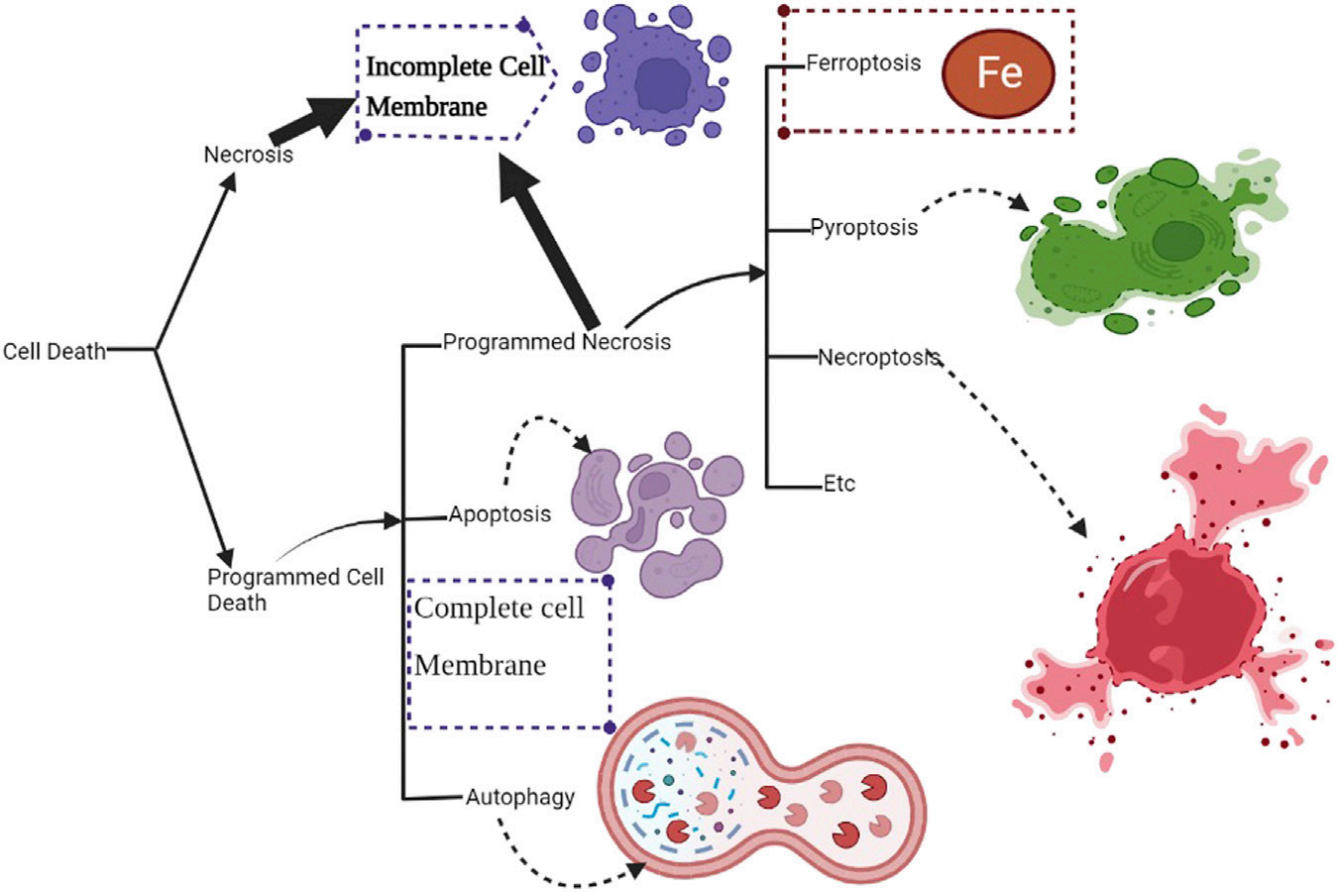
An illustration of p53’s role in ferroptosis. Cell death mediated by p53 is largely dependent on programmed necrosis. Programmed cell death mediates iron-dependent cell death, ferroptosis.

Van Maerken et al. (2013) have postulated that p53 is inactivated when MDM2 is activated. Saraf et al. have cited p53 mutation as the regulation of atypical ferroptosis, whereas (Komori, 2016) have indicated that interference with the morphology of p53 disrupts normal cell physiology and function. Impaired cell physiology undermines the interaction between SLC7A11 and p53, leading to enhanced tumor development. Additionally, a review of the outcomes reported by the two studies has indicated possible up- and downregulation of atypical ferroptosis in osteosarcoma. Moreover, p53 inactivation, as reported by the studies, undermines the formation of the SLC7A11-p53 complex, promoting osteosarcoma. The converse of this phenomenon is true, as p53 activation would not promote tumorigenesis. Figure 8 is a schematic representation of the floated p53 activation; Table 5 corroborates the scope provided in the Figure 8 by indicating that cell death is downregulated when osteoblast proliferation is initiated. The latter reflects p53’s down-regulatory function achieved when the processes enhancing proliferation are down-scaled. In addition, the upregulation of p53 directly antagonizes the activity of SLC7A11 (Liu et al., 2022). The direct antagonism of SLC7A11 enhances ferroptosis in osteosarcoma (Table 6).

**TABLE 6:** A summary of p53’s roles in typical and atypical ferroptosis in osteosarcoma.

P53 regulatory roles in typical ferroptosis	P53’s regulatory roles in atypical ferroptosis
Protein-protein interaction with SLC7A11	Inactivation of p53 through MDM2 activation
Modulation of target genes	Genetic mutations that alter cellular functions
Down- or upregulation regulation of tumorigenesis	Production of reactive oxygen species
Initiation of p53’s antitumor functions	Regulatory effects of mutant-p53

## Discussion

The regulatory role of p53 in atypical and typical ferroptosis in osteosarcoma remains unknown. However, the regulatory effects of p53 are rapidly evolving, and many scholars have studied its role. In the present study, we aimed to unmask the role of p53 in ferroptosis. Several findings arose in the present study, implicating clinical significance. They included the up- and downregulation of ferroptosis in both atypical and typical ferroptosis in osteosarcoma. In addition, we found that either of the two regulatory roles enhanced ferroptosis, resulting in tumor progression.

**FIGURE 9:**
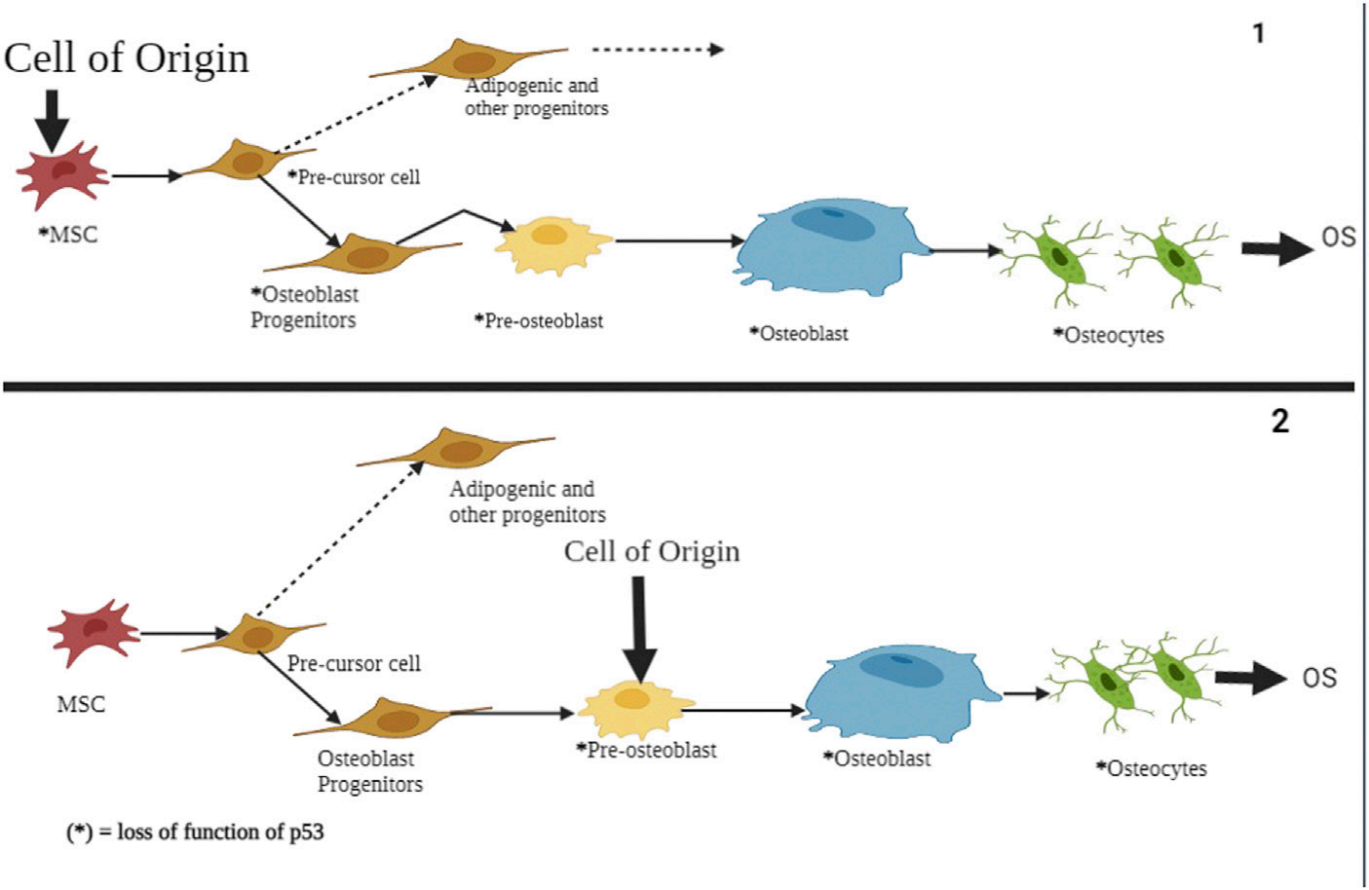
A schematic representation of p53’s role in atypical and typical ferroptosis in osteosarcoma. In both cases, the functional loss of p53 is the origin of osteosarcoma and undifferentiated MSCs. The regulatory mechanisms affect osteoblasts, pre-osteoblasts, and undifferentiated MSCs. Mutation of the p53 tumor suppressor genes results in aberration of pre-osteoblast cells and mesenchymal progenitor cells, subsequently showing proliferation and compromised growth alongside terminal differentiation.

Since osteosarcoma is a severe health issue for adults and children, it is urgently necessary to comprehensively understand various biological processes underpinning its occurrence. Among the multiple biological agents, the regulatory role of p53 is involved in ferroptosis associated with osteosarcoma. The literature on osteosarcoma suggests that p53 facilitates up- and downregulation of ferroptosis, as shown in Figure 9. Interestingly, up- and downregulation of ferroptosis implicate outcomes of great clinical significance. 

Typical ferroptosis in osteosarcoma remains a function of different elements and biological processes within the cells. After evaluating evidence from 10 studies (Velletri et al., 2016; Luo et al., 2018; Hatina et al., 2019; Lu et al., 2020; Usman et al., 2020; Sciot, 2021; Magri et al., 2021; Ji et al., 2022), we found strong evidence on different regulatory frameworks of p53. Genetic regulatory outcomes stood out in the review as Ji et al., Luo et al., 2018, Lu et al., 2020, Velletri et al., 2016, Sciot, 2021, and Gnanapradeepan et al. have reported strong genetic evidence in regulatory processes of p53. A general observation of the outcomes reported on typical ferroptosis indicates strong up- and downregulation of ferroptosis.

According to Ji et al., the target genes of p53 are fundamental in the regulation of typical ferroptosis in the development of osteosarcoma. Furthermore, tumor suppression or progression is a function of p53 to select cells upon stimulation or inhibition through protein or gene expression. This finding is supported by several studies (Hatina et al., 2019; Usman et al., 2020; Magri et al., 2021), where a direct modulation of target cells involved in or modulating tumor cells has been reported.

Lu et al. (2020) have mentioned that PVT1 and miRNA-214 are critical players in the regulatory effects of p53 on osteosarcoma-related ferroptosis. PVT1 inhibits miRNA-214 overexpression, which leads to minimal or poor miRNA-214-p53 binding. The latter reduces ferroptosis as the functions of p53 are significantly reduced. A somewhat similar phenomenon unfolds through p53 activation, where the gain-of-function mutant implicates oncogenic functions. The p53 mutant is an agent of altered functions in ferroptosis events as it reverses the normal biological effects, increasing the gene expression and enhancing consequential ferroptosis.

Usman et al. and Magri et al. have reported that the p53 mutant mediates the down-modulation of xCT, a tumor-linked antigen that protects cells against ferroptosis and oxidative stress. The additional functions of xCT, such as metabolic reprogramming, chemoresistance, and enhanced tumor progression, unmasks that p53 plays a role in regulating ferroptosis in osteosarcoma. Magri et al. have reported that p53 downregulates xCT, resulting in enhanced tumor progression and metabolic reprogramming. These outcomes represent adverse outcomes of p53 and regulatory effects of ferroptosis in osteosarcoma. The literature on genetic mutations and ferroptosis reveals the alteration of genes associated with osteosarcoma. The resulting abnormal cell growth and progression are attributed to the generated cellular defects. Figure 6 illustrates alterations in the deoxyribonucleic acid, suggesting altered cell functions. Theoretically, altered cell functions are central to ferroptosis through mechanisms like the production of ROS.

However, the pathogenesis of osteosarcoma remains unclear. The tumor suppressor, p53, has been associated with the prevention of tumorigenesis. Inactivation of this tumor suppressor enhances drug resistance and promotes the development of osteosarcoma (Chen et al., 2021). In addition, p53 has a context-dependent function in the modulation of lipid peroxidation in ferroptosis in three ways: inhibiting SLC7A11 expression, alongside promoting GLS2 and SAT1 expression (Kang et al., 2019). Lipid peroxidation and ROS production in amounts exceeding the detoxification rate are vital processes responsible for ferroptosis in many cells. The ROS interacts with essential macromolecules like proteins and lipids, resulting in cell death. In addition, lipid peroxidation produces unstable compounds (Liu and Gu, 2022a). The regulatory effects of p53 on typical ferroptosis in osteosarcoma are associated with protein-protein interaction where MDM2-p53 interaction emerges. Table 5 summarizes the mechanisms through which p53 inactivation is achieved, including MDM2-p53 interaction. Our findings were consistent with the existing literature (Neochoritis et al., 2014). This regulatory mechanism is a two-sided phenomenon, as p53 can be activated when MDM2 is inactivated. However, MDM2 inactivation suppresses the p53-MDM2 interaction, which does not result in cell proliferation. Figure 1 and Table 4 and Table 5 represents MDM2’s fuantagonistic effects on p53 protein. Upregulation of either typical or atypical ferroptosis is subject to MDM2’s influence on the protein. Activation by MDM2 enhances p53’s up-regulatory functions, and the converse is true.

Pang et al. (2020) have contested that TP53 mutation is the driver of the high incidence of osteosarcoma, indicating the critical subject in p53’s regulatory effects on atypical ferroptosis in the disease. The literature on p53 biology and osteosarcoma treatment emphasizes the oncogenic functions of PT53: maintenance of the proliferation of tumor cells and promoting tumor growth (Synoradzki et al., 2021). Since TP53 gives instructions for p53 production, its mutation threatens the latter process, implying decreased ferroptosis. This phenomenon provides the connection between p53 and ferroptosis, as suggested by previous studies (Liu et al., 2022). As summarized in Table 5, genetic mutation, TP53, and p53 alterations are critical factors in the progression of osteosarcoma. Mutated forms of these genes do not interact with their target sites, causing tumor progression. Our findings were consistent with a previous study in which p53 alterations fuel the progression and spread of tumors (Magri et al., 2021). Additionally, we found that MDM2 played a significant role in the inactivation of p53, and the activation of the former activates the latter, resulting in tumor progression.

Even so, the primary mechanism of p53 is binding to SLC7A11, a promoter of tumorigenesis, and inhibiting its expression. This process modulates the sensitivity and metabolism of cancer cells to ferroptosis (Zhang et al., 2022b). Even though the authors report atypical regulatory accounts, events leading to ferroptosis in the course of osteosarcoma are key. Several studies (Yang and Zhang, 2013; Van Maerken et al., 2014; Komori, 2016; Leroy et al., 2017; Saraf et al., 2018; Pang et al., 2020) have reported a downregulated atypical ferroptosis through inactivation. The theoretical perspectives explain this phenomenon by indicating that the inactivation of p53 hinders its binding and interaction with SLC7A11, resulting in the inhibition of SLC7A11 (explanation for Table 6). As a promoter of tumorigenesis, inhibition of SLC7A11 downregulates the progression of osteosarcoma (Zhang et al., 2018). The above-stated regulatory mechanisms on atypical ferroptosis can be reversed when p53 is activated. The activated version of p53 represses SLC7A11 and subsequently enhances ferroptosis. This postulation is consistent with Liu et al. that the upregulation of p53 enhances ferroptosis by inhibiting SLC7A11 functions (de Azevedo et al., 2019). Upregulation of ferroptosis is detrimental in osteosarcoma as signs will appear through bone fractures, inability to support the body posture, and maintenance of balance.

We found evidence opposing the outcomes of p53 inactivation in ferroptosis in the course of osteosarcoma. Several studies (Bouchalova et al., 2014; Xie et al., 2016; Gnanapradeepan et al., 2018; Babamohamadi et al., 2022; Su et al., 2022) have contended that p53 plays a central role in up-regulating atypical ferroptosis in the course of osteosarcoma through increasing the production of ROS, lipid and iron-mediated cell death, and cell proliferation (Table 4) (Liu and Gu, 2022b). Upregulation of atypical ferroptosis unfolds through increased production of ROS, lipid peroxidation and enhanced cell proliferation. High lipid peroxidation and ROS production intoxicates cells and disrupts cell physiology, leading to mass cell death. Subsequently, impaired cell physiology interferes with cell growth processes, including proliferation. Metastasis, alongside other complications, such as hypercalcemia, cancer cachexia, bone pain induced by cancer, metastasis of the epidural spinal cord compression, and pathological bone fracture, stand out as common complications (Brown et al., 2017; Eaton et al., 2021). The clinical significance of this regulatory role concerns the management of osteosarcoma. Management strategies seek ways of down-regulating p53 or inactivation to prevent tumor progression. 

## Conclusion

Even though osteosarcoma is an uncommon type of cancer and accounts for approximately 3% of diagnosed cancers, the severe outcomes due to its exacerbation and progression in a considerable population raise the alarm. Furthermore, the origin and cause of osteosarcoma remain unknown, making the development of an effective treatment or intervention an uphill task. Therefore, we systematically reviewed studies reporting the role of p53 in atypical and typical ferroptosis to unmask the biological processes and events characterizing osteosarcoma. Our findings provided valuable insights into clinical practice and drug development for effective medication.

We found that typical and atypical ferroptosis enhanced tumor progression in osteosarcoma, and p53 played fundamental modulatory roles. Furthermore, biological processes underscored the regulatory functions of p53: multiple events and biological processes characterized the outcomes of osteosarcoma. Figure 1 summarizes the biological processes underscoring p53’s functions; upregulation of the protein enhances typical and atypical ferroptosis. Activation by MDM2 up scalesferroptosis.

Protein interaction and its influence on target genes was a fundamental observation in the present review. In addition, p53 inactivation was a significant inhibitor of atypical ferroptosis as all the processes triggered or stimulated by the protein were hindered. Likewise, p53-associated processes that downregulated tumorigenesis were negatively affected by the activation of the protein. The SLC7A11-p53 interaction, which downregulated osteosarcoma, was negatively affected by p53 inactivation, and the converse was true. Liu et al. have contended that upregulation of p53 promotes ferroptosis through inhibiting SLC7A11.

The review reported the effects of MDM2 on p53-associated ferroptosis in osteosarcoma. We found that the interaction between MDM2 and p53 inhibited cell proliferation, slowing down tumorigenesis. MDM2-p53 interaction was of clinical significance as it could be used as an intervention against osteosarcoma. Table 5 summarizes p53 inactivation following MDM2 activation. Interaction between p53 and genes promotes typical or atypical ferroptosis to up- or downregulate osteosarcoma. We did not find up- and downregulation of the tumors occurring concomitantly.

Mutation and the effects of the mutant gene play a fundamental role in typical and atypical ferroptosis. The target genes and genetic expression underpinned the regulatory effects of p53 in osteosarcoma. Whichever direction is taken by the genetic and expression activities, osteosarcoma either increases or decreases. Several studies (Velletri et al., 2016; Luo et al., 2018; Hatina et al., 2019; Lu et al., 2020; Usman et al., 2020; Sciot, 2021; Magri et al., 2021) have reported the direct modulation of target genes and the effects of mutant-p53 in the tumor development process. We could take away that genetic mutations enhanced typical ferroptosis in osteosarcoma due to resulting changes in the deoxyribonucleic acid and cellular activities. The major takeaway from the mutant p53 gene concerns osteosarcoma progression based on the protein involvement. The genes are tailored to initiate tumorigenesis.

Additionally, we found that p53 complicated osteosarcoma management by inducing chemoresistance through the genetic pathways and processes. Genetic mutations, especially the mutant-p53, altered cell structures and functions, resulting in chemoresistance and enhancing cellular susceptibility to ferroptosis. With chemoresistance, interventions against osteosarcoma will be repelled, giving room to the progression of tumors and spreading to other cells. This phe nomenon bears much weight concerning the management of ferroptosis in osteosarcoma. The latter was a significant event leading to enhanced tumorigenesis. However, activation of p53 downregulated tumorigenesis and improved the health of osteosarcoma patients. 

Cell proliferation and inhibition of cell division are mechanistic models through which p53 downregulates ferroptosis. Several studies (Yang and Zhang, 2013; Van Maerken et al., 2014; Komori, 2016; Leroy et al., 2017; Saraf et al., 2018; Schiavone et al., 2019; Pang et al., 2020) have reported that the inactivation of p53 inhibits cell proliferation and subsequently downregulates atypical ferroptosis in osteosarcoma. Pang et al. have indicated that approximately 95% of osteosarcoma cases result from p53 inactivation.
